# Early cochlear implantation supports narrative skills of children with prelingual single-sided deafness

**DOI:** 10.1038/s41598-023-45151-x

**Published:** 2023-10-19

**Authors:** Tine Arras, An Boudewyns, Ingeborg Dhooge, Andrzej Zarowski, Birgit Philips, Christian Desloovere, Jan Wouters, Astrid van Wieringen

**Affiliations:** 1 Department of Neurosciences, Experimental ORL, KU Leuven, O&N2, Herestraat 49 Bus 721, 3000 Leuven, Belgium; 2 Cochlear Technology Center, Schaliënhoevedreef 20i, 2800 Mechelen, Belgium; 3 Department of Otorhinolaryngology, Antwerp University Hospital, Drie Eikenstraat 655, 2650 Edegem, Belgium; 4 Faculty of Medicine and Translational Neurosciences, University of Antwerp, Universiteitsplein 1, 2610 Wilrijk, Belgium; 5 Department of Otorhinolaryngology, Ghent University Hospital, C. Heymanslaan 10, 9000 Ghent, Belgium; 6 European Institute for ORL-HNS, Sint-Augustinus Hospital Antwerp, Oosterveldlaan 24, 2610 Wilrijk, Belgium; 7 Department of Otorhinolaryngology, Head and Neck Surgery, University Hospitals Leuven, Herestraat 49, 3000 Leuven, Belgium

**Keywords:** Paediatric research, Auditory system

## Abstract

Prelingual single-sided deafness (SSD) not only affects children’s hearing skills, but can also lead to speech-language delays and academic underachievement. Early cochlear implantation leads to improved spatial hearing, but the impact on language development is less studied. In our longitudinal study, we assessed the language skills of young children with SSD and a cochlear implant (CI). In particular, we investigated their narrative skills in comparison to two control groups: children with SSD without a CI, and children with bilateral normal hearing. We found that children with SSD and a CI performed in line with their normal-hearing peers with regard to narrative and verbal short-term memory skills. Children with SSD without a CI had worse narrative (group difference = − 0.67, p = 0.02) and verbal short-term memory (group difference = − 0.68, p = 0.03) scores than the implanted group. Verbal short-term memory scores and grammar scores each correlated positively with narrative scores across all groups. Early grammar scores (at 2–3 years of age) could partially predict later narrative scores (at 4–6 years of age). These results show that young children with prelingual SSD can benefit from early cochlear implantation to achieve age-appropriate language skills. They support the provision of a CI to children with prelingual SSD.

## Introduction

Children with congenital single-sided deafness (SSD) are born with profound hearing impairment (HI; > 90 dB HL) in one ear, and normal hearing (NH) in the contralateral ear. The resulting absence of binaural hearing causes poor sound localization and speech perception in noise^1^. In addition, children with SSD are at risk for various developmental difficulties^2^, including impaired balance skills^3^ and speech-language delay^4–6^. Untreated SSD may also cause cortical reorganization in favor of the NH ear, known as the aural preference syndrome^7^.

Providing a cochlear implant (CI) to children with SSD can partially restore their bilateral sound perception and might enable binaural hearing. Cochlear implantation is associated with improved sound localization and speech perception in noise in adults with acquired SSD^8–10^ and children with SSD^11–13^. For children with congenital SSD, early cochlear implantation, during a period of high neuroplasticity, promotes optimal auditory outcomes^14^ and may prevent or reduce the aural preference syndrome^15^. In an earlier study, we found that the CI also supported normal early grammar development in children with prelingual SSD^16^, but the impact on other aspects of language remains unclear.

While children with prelingual SSD are at risk for poorer early language skills, little is known about the impact of SSD on the later development of language. On one hand, unilateral HI has been shown to negatively affect general language scores in adolescents^17^. On the other hand, poor early language development likely has a cascading effect on children’s later proficiency in more complex language skills. For example, oral narrative comprehension relies on multiple language and cognitive skills, including verbal working memory, grammar, vocabulary, inference, and theory of mind^18–20^. To understand a story, a listener must temporarily store the speech information, extract the relevant syntactic structures and words, and attach meaning to them. Successful inference making and theory of mind help the listener understand why certain events take place. For storytelling, the corresponding expressive ability, similar skills are required. Narrative skills expand gradually throughout childhood^21^, and are interrelated with other aspects of language. Early grammar abilities can partially predict the later narrative skills of children^22^. In addition, vocabulary knowledge seems to be associated with both narrative comprehension^19^ and shared reading^23^. In turn, early childhood storytelling competence may be predictive for concurrent (i.e., measured at the same age) or later academic achievement, including literacy and math^24–27^.

Research regarding the narrative skills of children with unilateral HI is limited. Children with unilateral HI may have poorer verbal narrative comprehension in challenging listening conditions^28^ as well as delayed expressive narrative development^29^. Children with bilateral HI, on the other hand, produce oral narratives with less lexical diversity, lower morphosyntactic complexity and more errors compared to those of their normal hearing peers^30–33^. In addition, they often achieve lower scores on story-retelling tasks^34–37^.

When retelling a story, verbal short-term memory (STM) likely plays an important role as well, given the need to remember the story content and specific words and phrases. Verbal STM refers to the capacity to accurately store and retrieve verbal information. For example, when learning new words, children must retain the phonological representation of those words long enough to assign meaning to them. Indeed, verbal STM capacity is associated with vocabulary knowledge in young normal-hearing children^38–41^. Similarly, it is linked to better sentence processing^42^, higher grammar scores^43^ and improved listening comprehension, especially in noise^44^. In preschool children, word span scores can predict sentence repetition scores and the average utterance length in spontaneous speech^45^. Finally, verbal STM capacity is associated with story-retelling abilities^46^ and later reading skills^47–49^.

The impact of unilateral HI on verbal STM is largely unknown. A pilot study by Ead et al.^50^ found decreased non-word repetition scores in children with unilateral HI, and adults with unilateral HI seem to have shorter digit spans compared to NH peers as well^51^. Children with bilateral HI may also have reduced verbal STM capacity^52–54^. In children and adolescents who use CIs, digits spans are typically shorter compared to those of their NH peers^55–57^, and they are linked to the children’s speech comprehension and reading skills^58,59^. Early rather than late cochlear implantation seems to improve verbal STM performance^60^. Interestingly, even children with NH have shorter digit spans in the presence of noise^61^, suggesting that overall audibility affects verbal STM. Similarly, children with NH with parent-reported listening difficulties may have poorer verbal STM^62^. Based on these findings, it seems likely that children with SSD would also have reduced verbal STM capacities, and that early cochlear implantation could limit the impact of SSD on these skills.

Taken together, research suggests that children with prelingual SSD may be at risk for poorer narrative skills. The aim of the current study was to investigate the development of narrative skills in a group of young children with prelingual SSD with and without a CI. Given the positive impact of a CI on early grammar skills in children with prelingual SSD^16^, we expected that early cochlear implantation would support normal narrative development as well. In addition, we explored the association between narrative skills on one hand and verbal STM and grammar on the other hand across all groups. We expected that adequate verbal STM and grammar would enable accurate story retelling. Finally, we investigated if early grammar skills could predict later narrative abilities. We expected that children with strong early grammar skills would have good narrative skills at a later age.

## Methods

The current study was designed and conducted according to the Declaration of Helsinki. The medical-ethical committee of the University Hospitals Leuven reviewed and approved the study protocol (registration number: B322201523727). Written informed consent was obtained from the parents of all participants.

### Participants

All participants were enrolled in a longitudinal multicenter study. The children with SSD (n = 33) were recruited from four academic hospitals in Belgium between 2015 and 2020. At the time of inclusion, normal hearing thresholds in one ear (≤ 35 dB nHL) and severe-to-profound hearing loss in the contralateral ear (≥ 80 dB nHL) were confirmed using click-evoked auditory brain stem response audiometry. In all but two cases, the hearing loss was present at birth. Cochlear implantation was considered if children had an intact auditory nerve, confirmed by magnetic resonance imaging of the posterior fossa, and were younger than 2.5 years. As such, 18 children in the current study received a CI, with a mean age at implantation of 15.1 ± 5.6 months. Two of them experienced a progressive hearing loss in the better ear, for which they were fitted with a hearing aid. The remaining children with SSD (n = 15) were not implanted due to cochlear nerve deficiency (CND, n = 9), their age at the time of inclusion (> 2.5 years, n = 3), or lack of parental consent (n = 3). The control group consisted of 28 children with bilateral NH. All children were native Dutch speakers.

All children in the SSD + CI group received a Cochlear™ Nucleus® implant and a Nucleus or Kanso sound processor. On average, they wore their speech processor for 8.2 ± 2.7 h per day (range 2.5 to 11.9 h). The children received no specific rehabilitation within the framework of the study, although some of them received therapy on an individual basis if deemed necessary by their caregivers (e.g., speech-language therapy, physiotherapy). Additional child characteristics are presented in Table 1. Most participants in the current study also contributed data to our work on the early language skills of children with SSD^16^.

**Table 1:** Demographic information of the participants.

Characteristic no. (%)	SSD + CI group (n = 18)	SSD group (n = 15)	NH group (n = 28)	Total (n = 61)
Sex				
Female	8 (44)	6 (40)	11 (39)	25 (41)
Male	10 (56)	9 (60)	17 (61)	36 (59)
Laterality of hearing loss				
Left	7 (39)	9 (60)	NA	16 (48)
Right	11 (61)	6 (40)	NA	17 (52)
Cause of deafness				
Congenital cytomegalovirus infection	13 (72)	3 (20)	NA	16 (48)
Cochlear nerve deficiency	0	9 (60)	NA	9 (27)
Cochlear malformation (incomplete partition type II)	1 (6)	0	NA	1 (3)
Petrous bone fracture (at age 10 months)	1 (6)	0	NA	1 (3)
Meningitis	1 (6)	0	NA	1 (3)
Unknown	2 (11)	3 (20)	NA	5 (15)
Comorbidities				
Premature birth	2 (11)	1 (7)	0	3 (5)
Cerebral palsy	2 (11)	0	0	2 (3)
Oculo-auriculo-vertebral dysplasia	0	1 (7)	0	1 (2)
Maternal education level				
Secondary education (high school)	3 (17)	3 (20)	4 (14)	10 (16)
Higher education (academic or nonacademic)	15 (83)	12 (80)	24 (86)	51 (84)
Birth order				
First or only child	2 (11)	6 (40)	14 (50)	22 (36)
Second or later child	16 (89)	9 (60)	14 (50)	39 (64)

### Test materials

Once or twice per year, we administered various subtests from the Schlichting Test for Expressive Language^63^ (a Dutch language test) to children aged 2 to 6 years. These included the Story test, the Auditory Memory test, and the Sentence Development test. The assessments took place in a quiet room at the participants’ homes. The examiner provided verbal instructions and presented all test items using live voice. The children wore their devices using their everyday listening settings. Each test resulted in one age-referenced score for each assessment.

We assessed the narrative skills of children aged 3.8 years or older using the Story test. The participants listened to a short story before retelling it with picture support. They received points for repeating specific words or expressions (e.g., “promised”), for using certain grammatical structures (e.g., the use of “he says/thinks”), and for story complexity (e.g., using at least one verb per picture).

As proxy for verbal STM, we used the Auditory Memory test, which is suitable for children aged 2.8 years or older. The participants had to repeat word lists of increasing length, ranging from one to five words (e.g., “bus”, “chair nose table mouth eye”). Each correct list yielded one point.

Grammar skills were assessed using the Sentence Development test from the age of two. The participants had to repeat sentences (e.g., “also a fish, [also a fish]”), complete sentences (e.g., “a man with beard and a man [without beard]”), or adjust sentences (e.g., “this is for him and [these are for him]”). They received points for each correct utterance.

The test manual reports good reliability and concept validity for all tests. Lambda-2 coefficients for internal consistency equal 0.85 for narrative skills, 0.80 for verbal STM, and 0.90 for grammar. The correlations between raw score and age are 0.57 for narrative skills, 0.66 for verbal STM, and 0.78 for grammar^63^. Age-referenced scores are available in the test manual, based on one-month intervals.

### Statistical analysis

We used R version 4.2.0^64–71^ for data analysis and visualization. For all analyses, the significance level α was set to 0.05. Normal distribution of the results was assessed using Shapiro–Wilk tests.

Scores for each test were analyzed using a linear mixed model (LMM). This model accounts for the clustering effect due to repeated testing of the same subjects by adding the subjects’ identity as a random effect^72^. As covariates, we chose four variables that may influence language outcomes in young children: age, sex, maternal education level (MEL), and birth order (first- or later born). Given the use of age-referenced scores as outcomes, we did not expect to find any age effect in the results. Indeed, such an age effect would most likely reflect a learning effect due to repeated measurements with the same test materials. We included sex as a covariate, as some studies suggest that girls develop language skills more rapidly than boys^73,74^. Similarly, children from highly educated mothers typically perform better on language and other developmental tests^75,76^, which is consistent with some of our earlier findings in this group of children^16^. Finally, some studies suggest that firstborn children may have stronger expressive language skills than children with older siblings^77^.

The initial model included group as the main predictor variable; age, sex, MEL, and birth order (BO) as covariates; an interaction term for group and age; and subject as random effect: *score* ~ *group* + *age* + *sex* + *MEL* + *BO* + *group:age* + *1|ID.* For the subsequent model selection, we applied a backward elimination procedure. The significance of each fixed effect was calculated using type II Wald chi-square tests. Post-hoc comparisons were performed using Tukey pairwise tests with Bonferroni correction, based on the estimated marginal means from the LMM. Residual variances were compared using Levene’s test for homogeneity of variance.

To explore the connection between narrative skills and auditory memory, we compared the children’s scores for both tests at concurrent assessments (i.e., obtained at the same age) using Pearson’s correlations. We calculated the total and group-specific correlations between both measures. In a similar analysis, we calculated the correlation between the children’s grammar scores and narrative skills.

Finally, using the children’s early grammar scores, we created a linear model to predict their narrative scores. The model estimated the children’s average narrative score based on three predictors: their average “early” grammar score (between 2 and 3.7 years of age), their average score on the verbal STM task (across all assessments), and their participant group. Maternal education level was added as a covariate.

The full analysis related to grammar is available in the Supplementary files. This includes an analysis for all grammar scores (regardless of age), and a separate analysis for “early” scores (all scores between 2 and 3.8 years) and “late” scores (all scores obtained after 3.8 years).

## Results

### Narrative skills

Longitudinal narrative data were available for 57 children, aged 3.8 to 6.6 years, across 132 assessments: 18 children in the SSD + CI group (mean age 4.6 ± 0.6 years), 14 children in the SSD group (mean age 4.7 ± 0.5 years), and 25 children in the NH group (mean age 5.1 ± 0.7 years). The age-corrected test scores reflected the children’s narrative abilities and followed a normal distribution (W = 0.99, p = 0.54). The mean score across all assessments was 0.54 in the SSD + CI group, − 0.12 in the SSD group, and 0.48 in the NH group.

**Figure 1:**
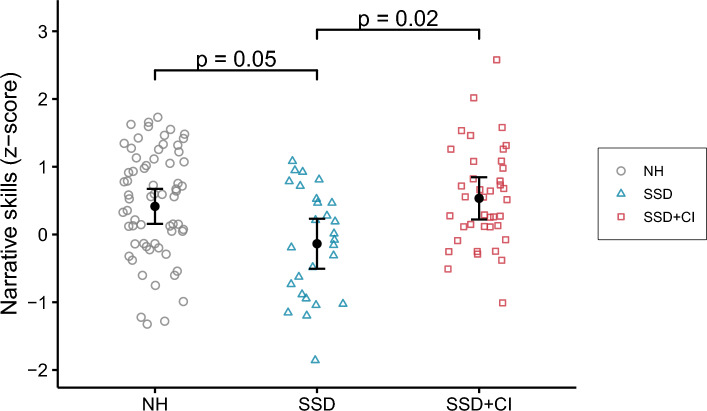
Individual standardized scores for the story test. The error bars represent the estimated marginal mean and corresponding standard deviation for each group.

Scores were significantly different across groups (X^2^ = 8.57, p = 0.01), with poorer scores in the SSD group compared to the NH group (− 0.55, p = 0.05) and the SSD + CI group (− 0.67, p = 0.02). The SSD + CI group had similar scores to those of the NH group (0.12, p = 1). None of the covariates had a significant effect on the scores. The model explained half of the variance (R^2^_m_ = 0.09, R^2^_c_ = 0.50), and the residual variances were similar across groups (F = 0.30, p = 0.74). The results are presented in Fig. 1. Individual developmental trajectories for all children with SSD, compared to the mean of the NH group, are available in Supplementary Fig. S1 (online). This figure illustrates that despite the overall poorer performance in the SSD group compared to both other groups, most of the children achieved age-appropriate scores (i.e., within one standard deviation from the population mean).

### Verbal short-term memory and its association with narrative skills

Longitudinal verbal STM data were available for 61 children, aged 2.8 to 6.8 years, across 212 assessments: 18 children in the SSD + CI group (mean age 4.1 ± 0.9 years), 15 children in the SSD group (mean age 4.1 ± 0.7 years), and 28 children in the NH group (mean age 4.5 ± 1.0 years). The age-corrected test scores quantified the children’s auditory memory span and followed a normal distribution (W = 0.99, p = 0.09). The mean score was 0.42 in the NH group, − 0.17 in the SSD group, and 0.36 in the SSD + CI group.

**Figure 2:**
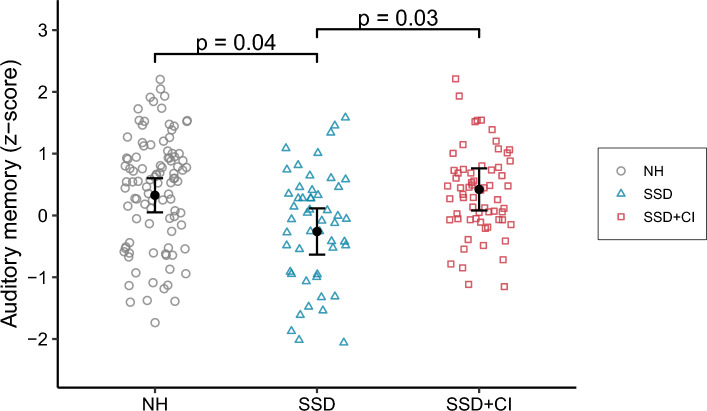
Individual standardized scores for the auditory memory test. The error bars represent the estimated marginal mean and corresponding standard deviation for each group.

Scores were significantly different across groups (X^2^ = 8.58, p = 0.01), with poorer scores in the SSD group compared to the NH group (− 0.59, p = 0.04). The children in the SSD + CI group outperformed their non-implanted peers (0.68, p = 0.03) and achieved scores similar to those of the NH group (0.10, p = 1). Again, none of the covariates had any significant effect on the scores. The model explained more than half of the variance (R^2^_m_ = 0.09, R^2^_c_ = 0.63), and the residual variances were similar across groups (F = 1.64, p = 0.20). The results are compared in Fig. 2. Individual developmental trajectories for all children with SSD are available in Supplementary Fig. S2 (online). For verbal STM, more children in the SSD group obtained scores below the normal range (i.e., more than one standard deviation below the mean).

**Figure 3:**
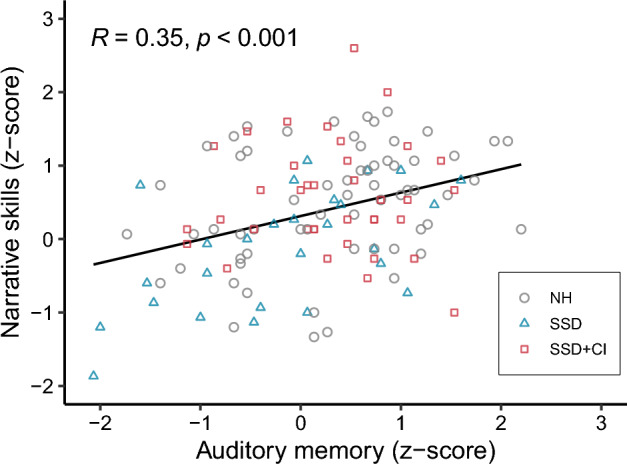
Correlation between auditory memory scores and narrative skills across all groups. The black line, with its corresponding R- and p-value on the top left, shows the correlation strength across all groups.

Concurrent results of the narrative and verbal STM tests (i.e., obtained during the same assessment) were available for 56 children, totaling 128 sets of results. Across all groups, a positive correlation was found between both measures (r = 0.35, p < 0.001). Children with large auditory memory spans typically had high scores on the narrative task (see Fig. 3). When comparing the correlation strength between groups, the strongest correlation was present within the SSD group (r = 0.56, p = 0.002). While there was a moderate correlation within the NH group (r = 0.32, p = 0.01), there was no correlation between auditory memory span and narrative skills in the SSD + CI group (r = − 0.01, p = 0.94). The correlation strength in each group is presented in Supplementary Fig. S3 (online).

### Association between grammar skills and narrative skills

With regard to late grammar skills (3.8 to 6.6 years), data were available for 57 children. The majority of these scores were previously reported in Arras et al.^16^. The scores were similar across groups, and there were no significant differences in the scores related to any of the other predictors.

**Figure 4:**
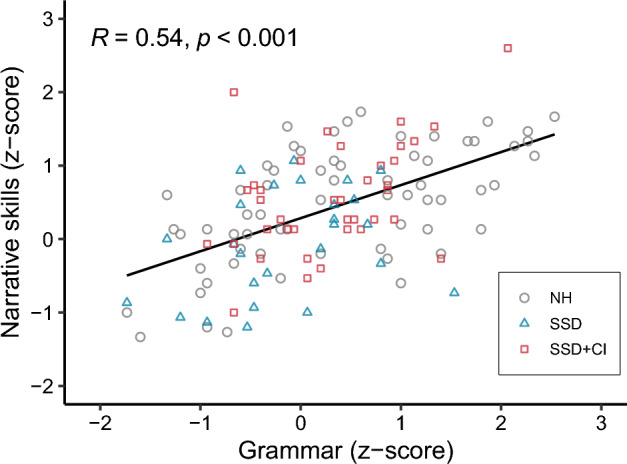
Correlation between grammar scores and narrative skills across all groups. The black line shows the overall correlation strength.

To investigate whether the children’s narrative skills were connected to their grammar abilities, we correlated their concurrent scores on each task. Concurrent results of the narrative and grammar tests were available from 56 children, for 128 sets of results. Across all groups, a positive correlation was found between both measures (r = 0.54, p < 0.001). This correlation was found in the NH group (r = 0.59, p < 0.001) and the SSD + CI group (r = 0.46, p = 0.004), but not in the SSD group (r = 0.34, p = 0.09) (see Supplementary Fig. S4, online). Children with strong grammar skills typically had high scores on the narrative task (see Fig. 4).

### Early grammar skills as predictor for later narrative skills

Data on early grammar skills (2 to 3.7 years) were available for 54 children. The majority of these scores were previously reported^16^. Scores were significantly different across groups (X^2^ = 12.69, p = 0.002), with poorer scores in the SSD group compared to the NH group (− 0.67, p = 0.003). The children in the SSD + CI group outperformed their non-implanted peers (0.51, p = 0.04) and achieved scores similar to those of the NH group (− 0.16, p = 1).

**Figure 5:**
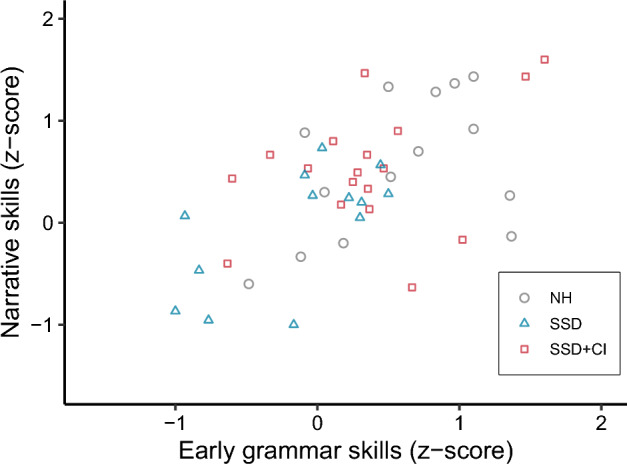
Average narrative scores as a function of early grammar skills.

Early grammar scores could predict the children’s later narrative scores with a coefficient of 0.60 ± 0.13 (F = 20.73, p < 0.001), as shown in Fig. 5. This indicates that children with high early grammar scores often had high scores on the narrative task a few years later. The other tested predictors (group, verbal STM, and maternal education level) were not significant. The model explained about one third of the variance (adjusted R^2^ = 0.31).

## Discussion

The current work describes the narrative skills of children aged 4–6 years with prelingual SSD with and without a CI. We found that children with SSD had slightly poorer scores than their NH peers on a story-retelling task, while children with SSD and a CI achieved scores that were similar to those of the NH group. In the same group of children, verbal STM was overall poorer in the SSD group, but not in the SSD + CI group. Across all groups, we found a positive correlation between narrative skills and both auditory memory span and grammar abilities. Finally, we found that early grammar skills could partially predict the children’s performance on the narrative task.

Previous research suggested that children with unilateral HI have poorer narrative skills^28,29^ and lower verbal STM capacity^50,51^. The current work matches these findings, as our group of non-implanted children with SSD had lower scores than their NH peers on both tasks. Yet, the majority of the children in the SSD group achieved age-appropriate scores on both tests. While their scores were significantly lower than those of their NH peers, most scores were within one standard deviation from the norm-based mean. This suggests that standard language tests may not be sensitive enough to detect subtle language deficits experienced by children with SSD. In addition, the normal variation in the language skills of young children may be a confounding factor.

We observed a significant correlation between auditory memory scores and story-retelling performance. A similar correlation was found earlier in a group of 4- and 5-year old children^46^. This association is not surprising, since having a large verbal STM capacity likely enables children to accurately reproduce a story. Alternatively, the combination of poor verbal STM and low narrative scores could reflect an underlying language deficit that affects all language-based assessments. Within the SSD + CI group, the correlation between auditory memory scores and narrative skills was not present. In this group, the range of scores on the auditory memory task was smaller than in both other groups, which could make it harder to detect the correlation.

Previous work showed that children’s grammar skills can predict their narrative abilities^22^. This association between grammar and narrative skills was also present in our data. The fact that early grammar skills (at 2–3 years of age) can have predictive value for the later development of advanced language skills is relevant from a rehabilitation perspective. This might allow for early identification of children with SSD who are at risk for language delays, and it facilitates targeted early intervention.

To the best of our knowledge, the current work is the first to investigate storytelling and verbal STM abilities in a group of children with SSD and a CI. In contrast to their non-implanted peers, our group of children with SSD and a CI achieved scores similar to those of their NH peers on both tasks. This suggests that early intervention with a CI supports normal development of narrative skills and auditory memory span. A possible explanation is that the CI enables improved speech perception and leads to decreased listening effort. This could in turn reduce the cognitive load when listening, leading to improved verbal STM capacity and better narrative comprehension. Subsequently, it would be easier to accurately recall and retell the story. An alternative explanation is based on earlier work showing that cochlear implantation supports early grammar development in children with SSD^16^. This improvement in early language skills may in turn support normal development of complex language skills at a later age, thus having a cascading effect on language outcomes throughout the child’s development. Alternatively, given that the current story-retelling task does not distinguish between points earned by content versus structure, the improved grammar abilities may have contributed directly to higher narrative scores.

The present work is among the first to describe narrative skills and verbal STM in children with SSD, as well as the impact of early cochlear implantation on these skills. The results should alert health care professionals to the risk for the subtle, but significant language deficits that children with SSD may experience. It is important to note that both narrative skills^24–26^ and verbal STM capacity^41,47,49^ in the last year of kindergarten have been linked to academic achievement in the first grade of primary school, including literacy skills. Therefore, even limited impairments or delays in the development of these skills may have a negative ripple effect on later developmental outcomes. As such, the current results might suggest that providing children with SSD with a CI not only improves their early language and hearing skills, but can even support their overall academic achievement. This should be taken into account when making health care decisions for these children.

For this study, an a priori power analysis indicated that the longitudinal design, in combination with LMM analysis, yielded sufficient power to detect the small but significant differences in performance between the groups. However, the study sample remains limited, with data collected from 61 children across three participant groups. As such, it is difficult to generalize these results to all children with prelingual SSD. As more children with SSD receive a CI, it will become easier for future studies to include more participants and draw conclusions that are more generalizable. Despite this limitation, our data support the provision of early cochlear implantation as standard of care in children with prelingual SSD.

## Conclusions

The current work explores the potential benefit of a CI on the complex language skills of young children with prelingual SSD. Our results suggest that early cochlear implantation supports the acquisition of narrative abilities and verbal STM. They extend our previous findings on early grammar skills in this population, where we found a significant difference between children with SSD with and without a CI. Early grammar skills, which can be measured at the start of kindergarten (at 2–3 years of age), seem to have some predictive value for the later development of more complex language skills, like storytelling. These findings affirm that cochlear implantation is an effective intervention to support the language development of children with prelingual SSD.

### Supplementary Information

Supplementary Information.

## Data Availability

The datasets generated during the current study are not publicly available due to privacy/ethical restrictions (as they contain information that could compromise the privacy of the participants). Anonymized data are available upon request from the corresponding author.
