# Supplementary material for: Early cochlear implantation supports narrative skills of children with prelingual single-sided deafness
Source: Sci Rep. 2023 Oct 19;13:17828. doi: 10.1038/s41598-023-45151-x (PMC10587124; doi:10.1038/s41598-023-45151-x)
Supplement: Supplementary file 1 — Supplementary Information. [file 41598_2023_45151_MOESM1_ESM.pdf]

Supplementary information for article "Early cochlear implantation supports narrative skills of children with prelingual single-sided deafness"

Authors: Tine Arras, An Boudewyns, Ingeborg Dhooge, Andrzej Zarowski, Birgit Philips, Christian Desloovere, Jan Wouters, Astrid van Wieringen

This Supplementary Information includes a number of supplementary figures (referenced in the main article), as well as a supplementary analysis of the grammar scores.

Part 1: Supplementary Figures

Supplementary Figure S1: Individual developmental trajectories of children with SSD for narrative skills, separated for the SSD+CI group (left) and the SSD group (right). The average score and standard deviation of the NH group for each age 6-month interval is shown in black. The dotted lines at +1 and -1 mark the cut-offs for the age-referenced "average" scores.

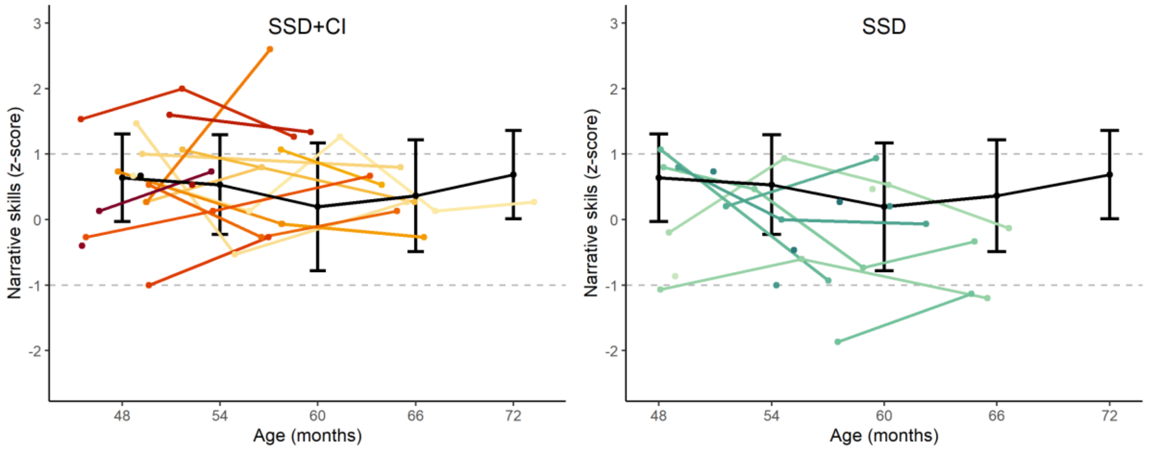

Supplementary Figure S2: Individual developmental trajectories of children with SSD for auditory memory, separated for the SSD+CI group (left) and the SSD group (right). The average score and standard deviation of the NH group for each age 6-month interval is shown in black. The dotted lines at +1 and -1 mark the cut-offs for the age-referenced "average" scores.

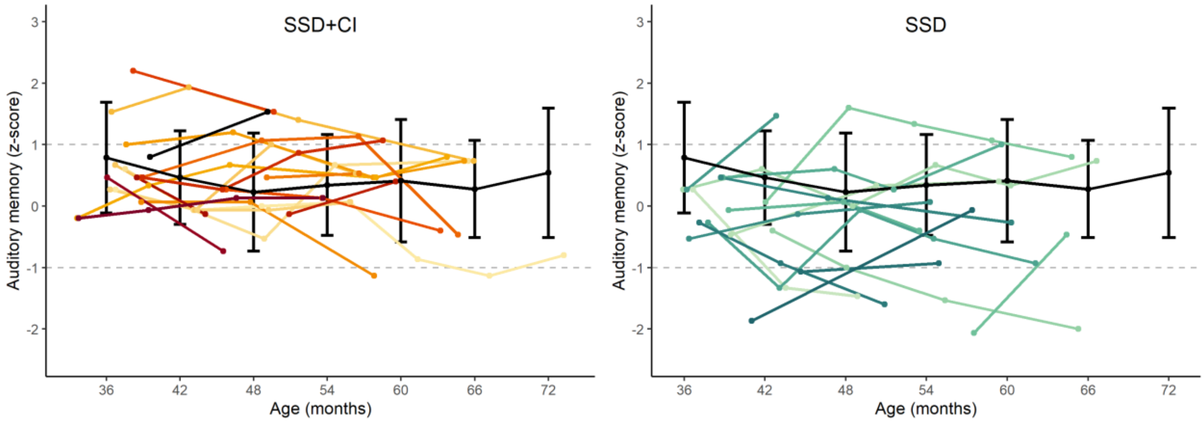

Supplementary Figure S3: Correlation between auditory memory scores and narrative skills within each group.

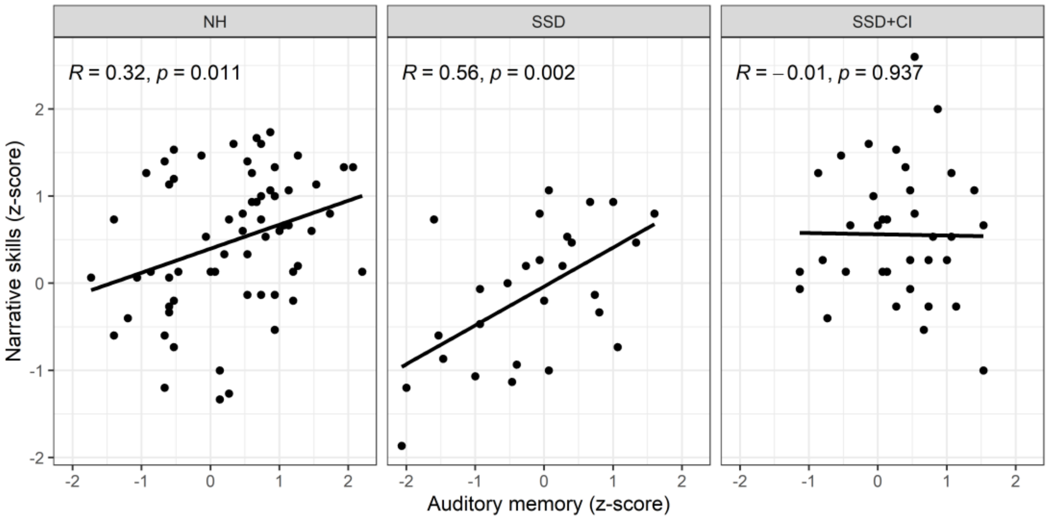

Supplementary Figure S4: Correlation between grammar scores and narrative skills within each group.

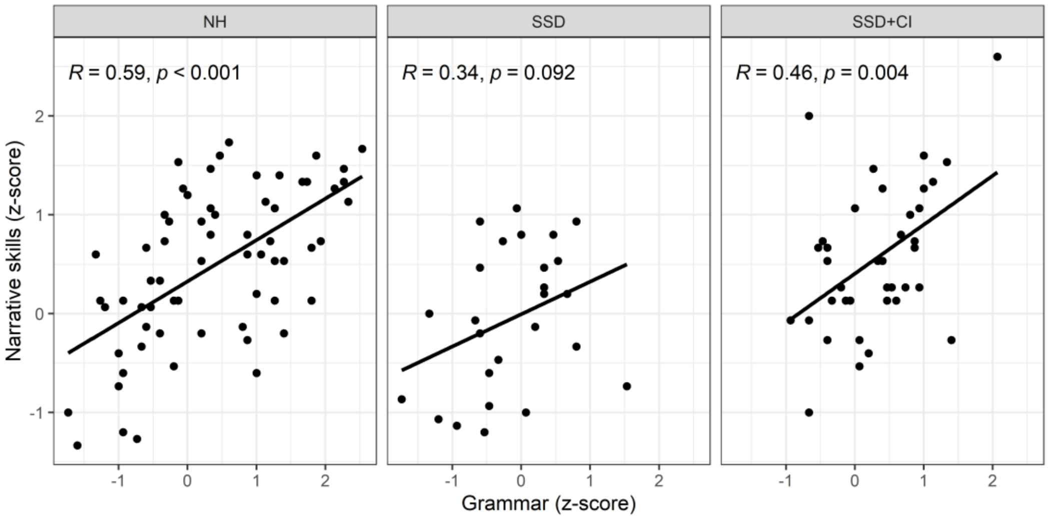

Part 2: Supplementary Analyses for grammar

Most of the grammar scores of the current participants have been discussed elsewhere (Arras et al., 2021). Therefore, they were not the focus of the current work. However, we did want to discuss the connection between early and later grammar scores on the one hand and narrative skills on the other hand. In this supplementary analysis, we provide more detail about the grammar scores as a separate outcome measure.

Longitudinal grammar scores

Longitudinal grammar data were available for 66 children, aged 2 to 6.6 years, across 292 assessments: 18 children in the SSD+CI group (mean age 3.6 ± 1.1 years), 16 children in the SSD group (mean age 3.6 ± 1.0 years), and 32 children in the NH group (mean age 3.9 ± 1.3 years). The age-corrected test scores quantified the children's grammar scores and followed a normal distribution (W = 1.00, p = 0.73). The mean score was 0.34 in the SSD+CI group, -0.16 in the SSD group, and 0.45 in the NH group.

Scores were significantly different across groups ($X^2 = 6.57$, p = 0.04), but the post-hoc contrasts were not significant after Bonferroni correction. Maternal education level was a significant predictor as well ($X^2 = 4.90$, p = 0.03), with children from mothers with higher education achieving higher grammar scores than their peers (0.47, p = 0.03). The model explained more than half of the variance (R^2_m = 0.11, R^2_c = 0.59), and the residual variances were similar across groups (F = 0.39, p = 0.68) and maternal education levels (F = 0.65, p = 0.42). The grammar scores are compared across groups (left) and maternal education levels (right) in Supplementary Fig. S5. Individual developmental trajectories for all children with SSD, compared to the mean of the NH group, are presented in Supplementary Fig. S6.

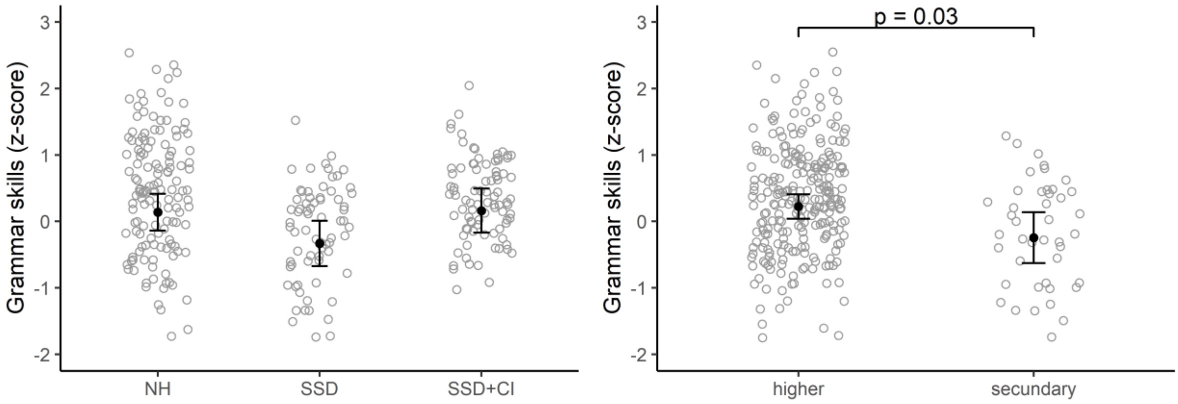

Supplementary Figure S5: Individual standardized scores for the grammar test, compared across groups (a) and maternal education levels (b). The error bars represent the estimated marginal mean and corresponding standard deviation for each group.

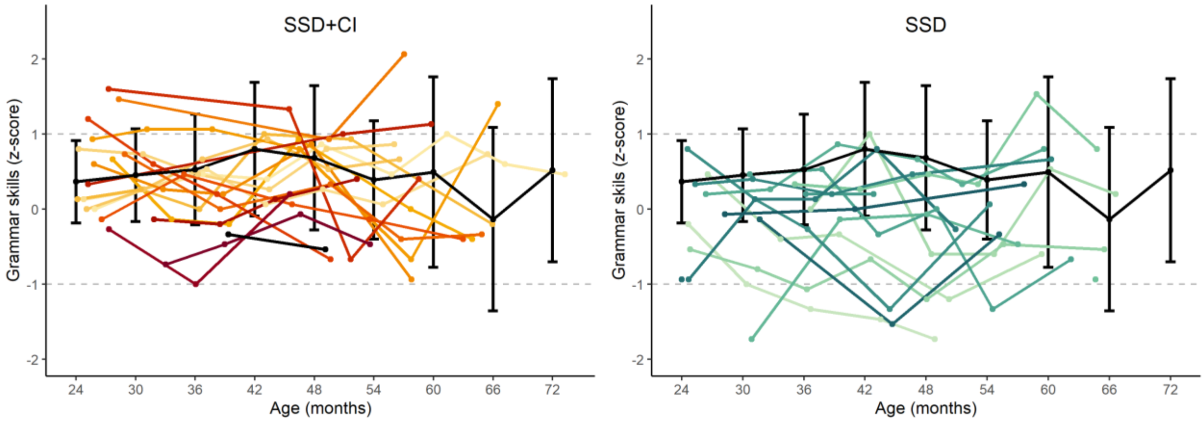

Supplementary Figure S6: Individual developmental trajectories of children with SSD for grammar, separated for the SSD+CI group (left) and the SSD group (right). The average score and standard deviation of the NH group for each age 6-month interval is shown in black. The dotted lines at +1 and -1 mark the cut-offs for the age-referenced “average” scores.

Early grammar scores

The subset of early grammar data, with scores of 54 children aged 2 to 3.7 years, spanned 156 assessments. This subset contained data for 18 children in the SSD+CI group (mean age 2.8 ± 0.5 years), 15 children in the SSD group (mean age 2.9 ± 0.5 years), and 21 children in the NH group (mean age 2.7 ± 0.5 years). Age-corrected test scores followed a normal distribution ($W = 0.99$, $p = 0.31$). The mean score was 0.33 in the SSD+CI group, -0.18 in the SSD group, and 0.52 in the NH group.

Scores were significantly different across groups ($X^2 = 12.69$, $p = 0.002$), with worse scores for the children with SSD compared to the NH control group (-0.67, $p = 0.003$). The children with SSD and a CI outperformed their non-implanted peers (0.51, $p = 0.04$) and achieved scores similar to those of the NH group. The model explained more than half of the variance ($R^2_m = 0.14$, $R^2_c = 0.60$), and the residual variances were similar across groups ($F = 2.34$, $p = 0.10$). The grammar scores for the three groups are compared in Supplementary Fig. S7.

As expected, these findings match our earlier report, which was based on (largely) the same dataset.

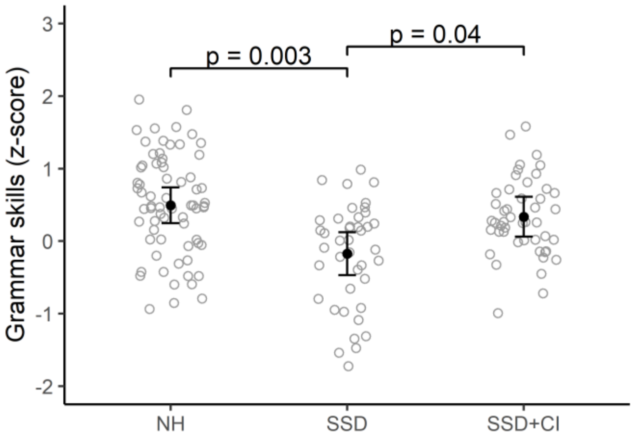

Supplementary Figure S7: Individual standardized scores for the early grammar test. The error bars represent the estimated marginal mean and corresponding standard deviation for each group.

Late grammar scores

The subset of late grammar scores contained data for 57 children aged 3.8 to 6.6 years, collected across 139 assessments. This included 18 children in the SSD+CI group (mean age 4.6 ± 0.6 years), 14 children in the SSD group (mean age 4.6 ± 0.5 years), and 25 children in the NH group (mean age 5.0 ± 0.4 years). Age-corrected test scores followed a normal distribution ($W = 0.99$, $p = 0.31$). The mean score was 0.35 in the SSD+CI group, -0.13 in the SSD group, and 0.39 in the NH group.

In this subset, no significant differences were found based on any of the predictors (group, age, maternal education level, and birth order), in contrast to the analysis of the full dataset and the early grammar scores. The most likely explanation for this difference is that the “late” grammar subset was not balanced enough. Of the 139 available records, 29 belonged to the SSD group, 41 to the SSD+CI group, and 69 to the NH group.
